# Supplementary material for: Sulfoquinovose Catabolism in E. coli Strains: Compositional and Functional Divergence of yih Gene Cassettes
Source: Int J Mol Sci. 2025 Oct 24;26(21):10351. doi: 10.3390/ijms262110351 (PMC12608037; doi:10.3390/ijms262110351)
Supplement: Supplementary file 1 [file ijms-26-10351-s001.zip › ijms-3902378-supplementary.pdf]

Sulfoquinovose Catabolism in *E. coli* Strains: Compositional and Functional Divergence of *yih* Gene Cassettes

Supplementary figures and tables

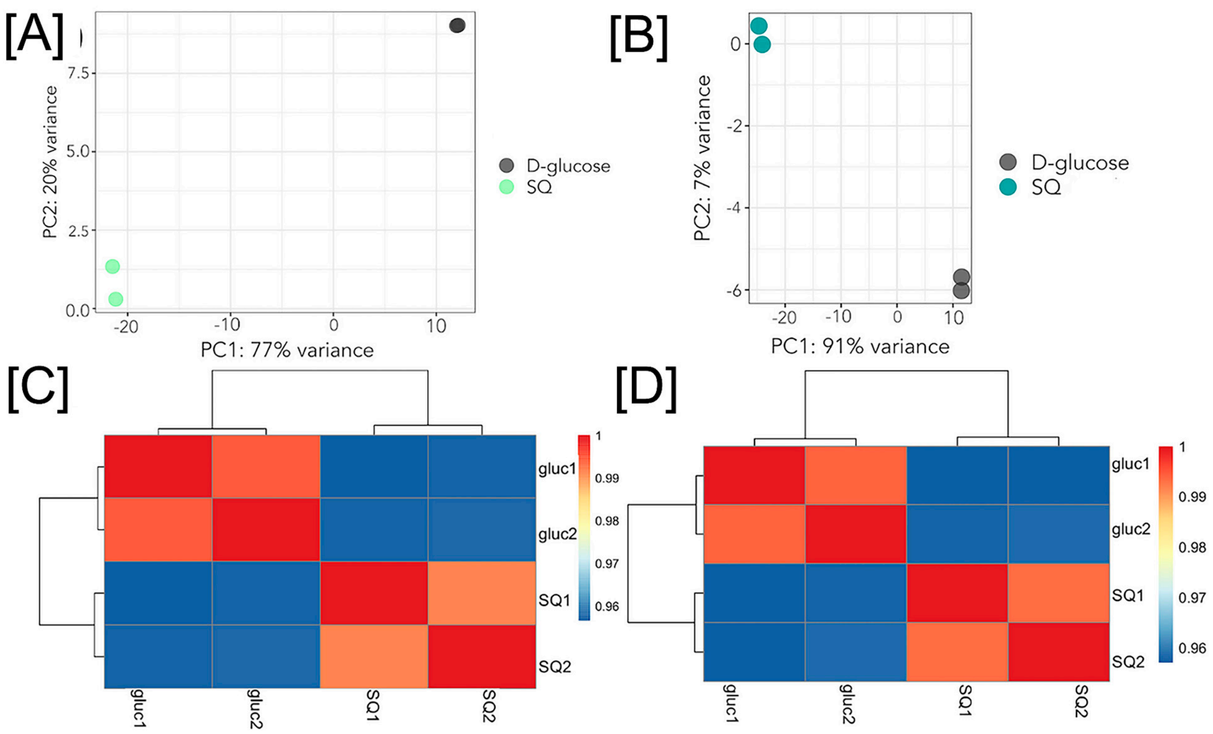

Supplementary Figure S1. Quality assessment of RNA-seq samples using PCA and hierarchical clustering based on regularized log (rlog) transformation of DESeq data. PCA plot for the *E. coli* K-12 MG1655 (A) and *E. coli* Nissle 1917 samples (B). Heatmaps showing pairwise correlation for the *E. coli* K-12 MG1655 (C) and *E. coli* Nissle 1917 samples (D).

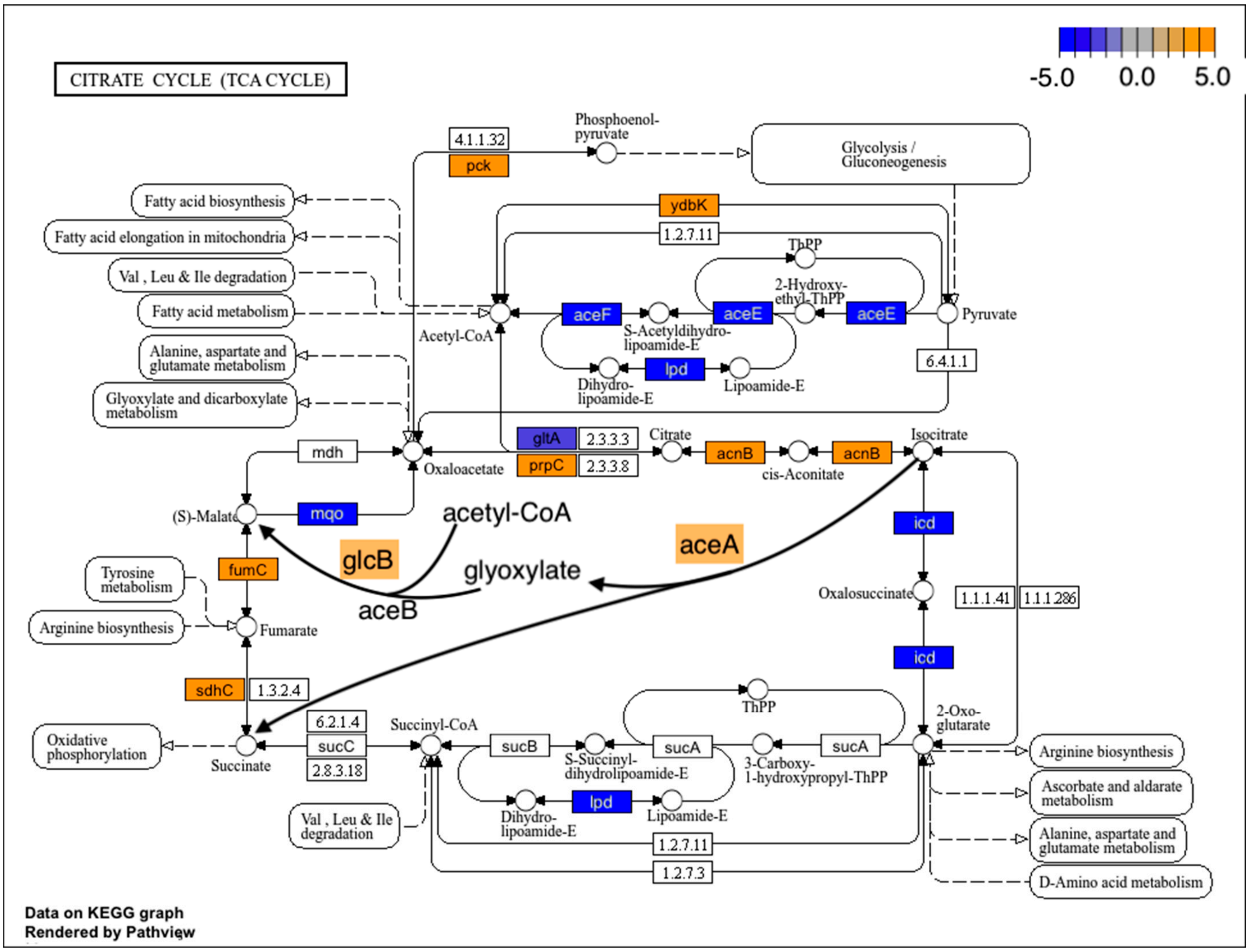

Supplementary Figure S2. KEGG pathway map of the central carbon metabolism in sulfoquinovose-grown *E. coli* K-12 MG1655 generated using pathview. Genes of the glyoxylate shunt (*glcB*, *aceA*, *aceB*) were manually added to the scheme. All genes are color-coded based on log_2(fold change) values compared to glucose-grown controls: blue indicates downregulation, orange indicates upregulation, and gray represents mid-range expression.

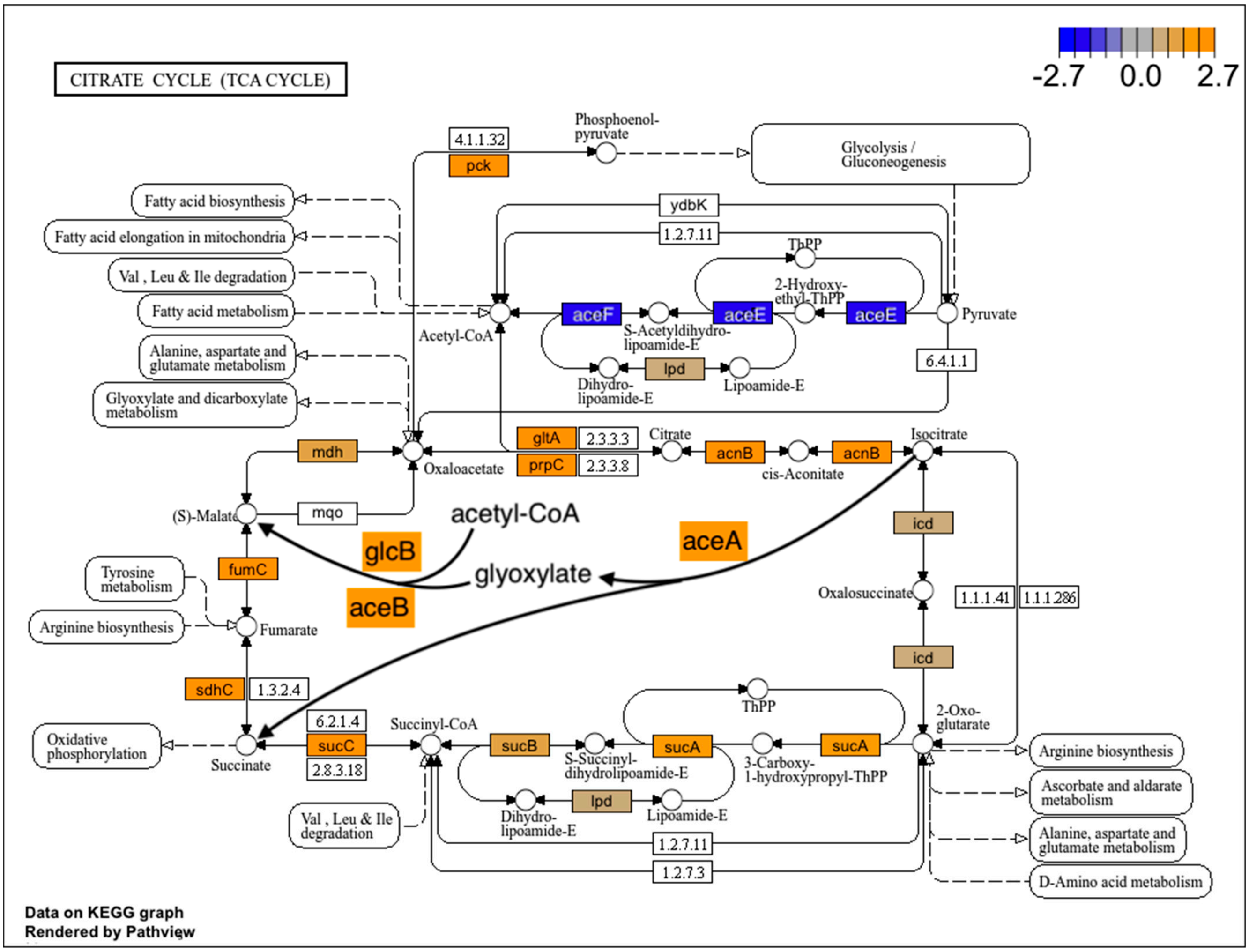

Supplementary Figure S3. KEGG pathway map of the central carbon metabolism in sulfoquinovose-grown *E. coli* Nissle 1917 generated using pathview. Genes of the glyoxylate shunt (*glcB*, *aceA*, *aceB*) were manually added to the scheme. All genes are color-coded based on $\log_2$(fold change) values compared to glucose-grown controls: blue indicates downregulation, orange indicates upregulation, and gray represents mid-range expression.

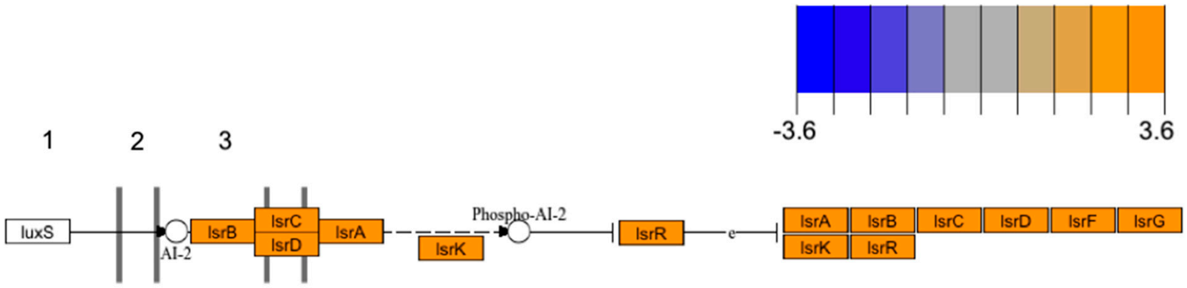

Supplementary Figure S4. KEGG pathway map of quorum sensing in sulfoquinovose-grown *E. coli* K12. Adapted from a figure generated with pathview. (1) Biosynthesis genes, (2) autoinducer, (3) sensing protein. Genes are color-coded based on $\log_2$(fold change) values compared to glucose-grown controls: blue indicates downregulation, orange indicates upregulation, and gray represents mid-range expression.

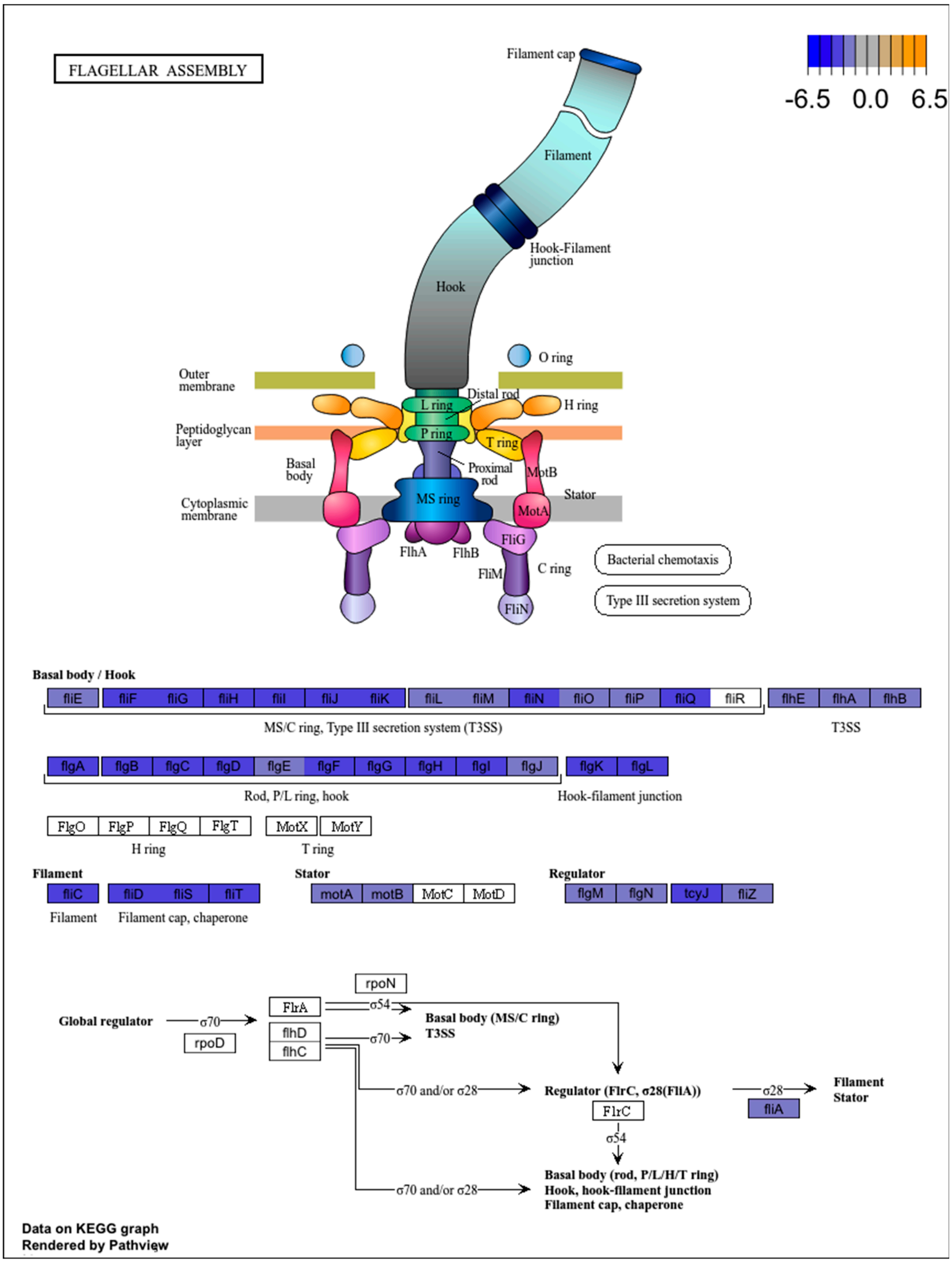

Supplementary Figure S5. KEGG pathway map of flagellar assembly in *E. coli* Nissle 1917. Gene expression of sulfoquinovose-grown *E. coli* Nissle 1917 was compared to glucose-grown controls. Flagellar synthesis genes are downregulated, shown in blue, while gray indicates mid-range expression.

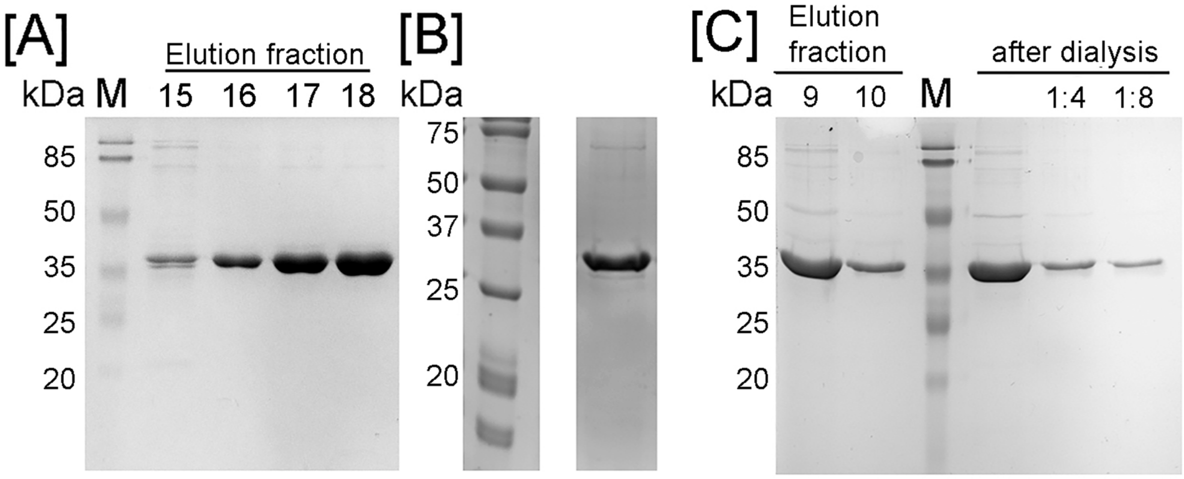

Supplementary Figure S6. Fraction of the purified YihW from *E. coli* K-12 MG1655 [A], and from *E. coli* Nissle 1917 [C]. Panel [B] represents the purified YihW from *E. coli* K-12 MG1655 after dialysis against a working buffer (see Materials and Methods section). 1:4 and 1:8 mean protein dilution in Binding Buffer (see Materials and Methods) prior to band-shift assays.

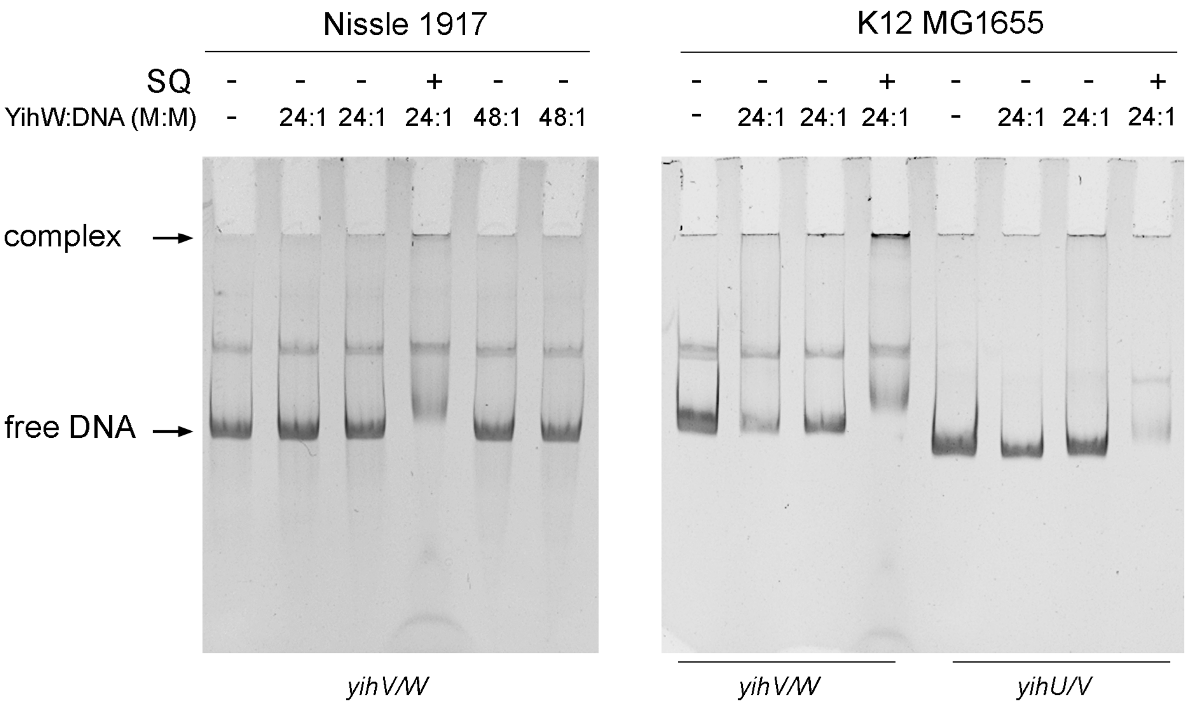

Supplementary Figure S7. Binding of YihW from *E. coli* Nissle 1917 to the *yihV/W* and *yihU/V* intergenic regions from *E. coli* Nissle1917 and *E. coli* K-12 MG1655 at 37°C. The YihW:DNA molar ratio, template strains, and the presence of SQ are indicated above the lanes.

Supplementary Table S1. Primers used in this study

**Primer name**	**Sequence (5’-3’)**
yihVW_F_uni	TYR ATG TGG TWG ATA CCA C
yihVW_R_uni	CAG ATK TGC CAG YTC WTC AAT
yihUV_F_uni	CAA TAR ATT GYT MGC CAT TGG
yihUV_R_uni	KGT KAT ACC YAC RCA WGC AA
yihW_PCR_uni	CMG ARC GYG GKT ATA TGA A
yihW_RT_uni	GCS GTA TTM AYG ACG CTG GA
yihV_PCR_uni	MGW RTT GCW TGY GTR GGT AT
yihV_RT_uni	AYG CGA CCR ATR AAA TCR AC
yihU_PCR_uni<br>yihU_RT_uni	ARC AAT YTA TTG CAR MAA GG<br>CCA TTS GGM ARC ATR GTR ATG
yihT_RT_uni	CAG AAY SGC MGA RGC ATA AG
yihT_PCR_uni	YGA RGC CAT GCG CMT GAT GT

yihP_PCR	CCG ACG CAA GAT CGG TCC A
yihP_RT	GAA CCA TTG CGC CGT AGG AG
yihR_PCR	TAT GCA TTG CAG TGG ACA GAC
yihR_RT	GGT TCG GCC ACG GAA TAA GC

Supplementary Table S2. Differentially expressed genes in sulfoquinovose-grown *E. coli* Nissle 1917 that are homologous to genes related to sulfoquinovose catabolism from Table S3. Operons were predicted and numbered with Operon-mapper (Taboada *et al.* 2018).

log2 Fold Change	locus_tag	gene	product	operon number	SQ catabolic protein	SQ catabolic gene	protein sequence identity, %	padj
-3.61	K8943_RS23555	*adhE*	bifunctional acetaldehyde-CoA/alcohol dehydrogenase	2427	ADE70656.1	*slaB*	24.359	1.38e-130
-0.57	K8943_RS07895	*fbaA*	class II fructose-bisphosphate aldolase	813	PRO65853.1	*sqiA*	27.063	6.85e-04
-2.12	K8943_RS17250	*aceE*	pyruvate dehydrogenase (acetyl-transferring), homodimeric type	1749	EJF39099.1	*sqwG*	39.583	6.57e-41
-1.74	K8943_RS12940	*ilvD*	dihydroxy-acid dehydratase	1301	WP_017967307.1	*sedC*	31.017	5.49e-15
-1.8	K8943_RS13765	*pfkA*	6-phosphofructokinase	1383	PRO65852.1	*sqiK*	27.597	2.68e-16

log2 Fold Change	locus_tag	gene	product	operon number	SQ catabolic protein	SQ catabolic gene	protein sequence identity, %	padj
-1.04	K8943_RS02045	*edd*	phosphogluconate dehydratase	256	WP_017967307.1	*sedC*	29.739	9.92e-03
-0.91	K8943_RS11115	*yhhX*	oxidoreductase	1109	MCD5344479.1	*sqgA*	35.556	1.48e-02
-0.75	K8943_RS00630	*ydfG*	bifunctional NADP-dependent 3-hydroxy acid dehydrogenase/3-hydroxypropionate dehydrogenase YdfG	83	WP_017967310.1	*sedA*	28.962	1.33e-03
-0.75	K8943_RS05805	*tkt*	transketolase	609	EJF39090.1	*sqwH*	29.391	7.64e-03
-0.56	K8943_RS16670	*tal*	transaldolase	1701	ADE70661.1	*sqvA*	29.448	1.00e-02
0.76	K8943_RS07970	*tkt*	transketolase	817	EJF39090.1	*sqwH*	25.0	1.10e-03
0.79	K8943_RS00890	*ydgJ*	oxidoreductase	116	MCD5344479.1	*sqgA*	34.783	3.65e-02
0.92	K8943_RS22550	*fabG*	3-oxoacyl-ACP reductase FabG	2336	WP_017967310.1	*sedA*	32.51	1.05e-07
1.4	K8943_RS13270	*tkt*	transketolase	1334	EJF39090.1	*sqwH*	24.823	1.47e-02
1.18	K8943_RS18980	*dxs*	1-deoxy-D-xylulose-5-phosphate synthase	1951	EJF39090.1	*sqwH*	31.959	2.66e-05
1.54	K8943_RS05660	*ucpA*	SDR family oxidoreductase UcpA	602	WP_017967310.1	*sedA*	36.111	2.28e-08

log2 Fold Change	locus_tag	gene	product	operon number	SQ catabolic protein	SQ catabolic gene	protein sequence identity, %	padj
1.75	K8943_RS09345	*ygjR*	Gfo/Idh/MocA family oxidoreductase	972	MCD5344479.1	*sqgA*	26.174	4.39e-06
1.47	K8943_RS18530	*betB*	betaine-aldehyde dehydrogenase	1899	ADE70656.1	*slaB*	34.021	3.14e-16
2.13	K8943_RS08845	*gpr*	L-glyceraldehyde 3-phosphate reductase	913	AAK90112.1	*squF*	24.516	4.02e-04
2.48	K8943_RS14825	*rpiB*	bifunctional allose-6-phosphate isomerase/ribose-5-phosphate isomerase RpiB	1491	EJF39089.1	*sqwI*	34.014	9.52e-04
3.45	K8943_RS00150	*patD*	aminobutyraldehyde dehydrogenase	23	ADE70656.1	*slaB*	35.22	1.68e-17
4.73	K8943_RS11905	*aldB*	aldehyde dehydrogenase AldB	1208	ADE70656.1	*slaB*	32.996	1.15e-42
1.82	K8943_RS06545	*gabD*	NADP-dependent succinate-semialdehyde dehydrogenase	691	ADE70656.1	*slaB*	36.364	3.32e-08
5.68	K8943_RS00020	*aldA*	aldehyde dehydrogenase	4	ADE70656.1	*slaB*	35.491	5.27e-78
6.87	K8943_RS01510	*astD*	succinylglutamate-semialdehyde dehydrogenase	192	ADE70656.1	*slaB*	28.025	4.20e-50

Supplementary Table S3. Number of RNA-seq reads assigned to the *yih* genes of *E. coli* K-12 MG1655 and *E. coli* Nissle 1917 during feature counting, with both strains grown on glucose or SQ as the sole carbon source. Each growth condition is represented by two biological replicates.

geneID	strain	gluc1	gluc2	SQ1	SQ2
ompL	K-12 MG1655	0	0	25	13
yihO	K-12 MG1655	2	1	149	169
yihP	K-12 MG1655	1	3	204	212
yihQ	K-12 MG1655	3	1	676	699
yihR	K-12 MG1655	0	0	142	148
yihS	K-12 MG1655	7	4	547	612
yihT	K-12 MG1655	3	1	234	356
yihT	Nissle 1917	0	2	4	1
yihU	K-12 MG1655	1	2	152	193
yihU	Nissle 1917	1	2	1	5
yihV	K-12 MG1655	8	7	134	176

yihV	Nissle 1917	13	12	6	12
csqR (yihW)	K-12 MG1655	9	7	47	71
csqR (yihW)	Nissle 1917	37	35	25	37

Supplementary Table S4. Differentially expressed genes in *E. coli* K-12 MG1655 and *E. coli* Nissle 1917 grown on sulfoquinovose compared to glucose.

strain	geneID	log2FoldChange	locus_tag	product	COGgene	padj	locus_tag of homologous gene from other strain
K-12 MG1655	*trpE*	-7.29	b1264	anthranilate synthase subunit TrpE	COG0147	1.57e-20	K8943_RS23680
K-12 MG1655	*trpC*	-4.93	b1262	fused indole-3-glycerol phosphate synthase/phosphoribosylanthranilate isomerase	COG0134	3.51e-94	K8943_RS23670
K-12 MG1655	*trpD*	-5.79	b1263	anthranilate synthase subunit TrpD	COG0547	1.43e-65	K8943_RS23675
K-12 MG1655	*trpB*	-6.4	b1261	tryptophan synthase subunit beta	COG0133	3.35e-135	K8943_RS23665
K-12 MG1655	*trpA*	-6.12	b1260	tryptophan synthase subunit alpha	COG0159	2.46e-137	K8943_RS23660
K-12 MG1655	*ilvM*	-6.12	b3769	acetolactate synthase II subunit IlvM	COG3978	9.01e-04	K8943_RS12930
K-12 MG1655	*ilvD*	-3.54	b3771	dihydroxy-acid dehydratase	COG0129	4.31e-38	K8943_RS12940
K-12 MG1655	*ilvA*	-2.81	b3772	threonine deaminase	COG1171	1.31e-13	K8943_RS12945
K-12 MG1655	*ilvE*	-4.5	b3770	branched-chain-amino-acid aminotransferase	COG0115	1.05e-33	K8943_RS12935
K-12 MG1655	*bioA*	-4.53	b0774	adenosylmethionine-8-amino-7-oxononanoate aminotransferase	COG0161	3.26e-04	K8943_RS20515

strain	geneID	log2FoldChange	locus_tag	product	COGgene	padj	locus_tag of homologous gene from other strain
K-12 MG1655	*mqo*	-4.43	b2210	malate:quinone oxidoreductase	COG0579	7.30e-26	K8943_RS04670
K-12 MG1655	*ilvB*	-4.23	b3671	acetohydroxy acid synthase I subunit IlvB	COG0028	3.04e-68	K8943_RS12455
K-12 MG1655	*ilvN*	-4.08	b3670	acetohydroxy acid synthase I subunit IlvN	COG0440	5.58e-14	K8943_RS12450
K-12 MG1655	*uhpB*	-3.77	b3668	sensory histidine kinase UhpB	COG3851	1.81e-11	K8943_RS12435
K-12 MG1655	*thiF*	-2.87	b3992	sulfur carrier protein ThiS adenylyltransferase	COG0476	4.59e-33	K8943_RS14180
K-12 MG1655	*thiH*	-2.11	b3990	2-iminoacetate synthase	COG1060	1.75e-25	K8943_RS14165
K-12 MG1655	*thiG*	-2.73	b3991	1-deoxy-D-xylulose 5-phosphate:thiol sulfurtransferase	COG2022	8.17e-32	K8943_RS14170
K-12 MG1655	*thiE*	-3.36	b3993	thiamine phosphate synthase	COG0352	3.07e-41	K8943_RS14185
K-12 MG1655	*thiS*	-3.29	b4407	sulfur carrier protein ThiS	COG2104	1.71e-11	K8943_RS14175
K-12 MG1655	*thiC*	-4.12	b3994	phosphomethylpyrimidine synthase	COG0422	6.59e-110	K8943_RS14190
K-12 MG1655	*metE*	-3.94	b3829	cobalamin-independent homocysteine transmethylase	COG0620	1.77e-104	K8943_RS13240
K-12 MG1655	*tyrA*	-3.94	b2600	fused chorismate mutase/prephenate	COG1605	4.04e-31	K8943_RS06430

strain	geneID	log2FoldChange	locus_tag	product	COGgene	padj	locus_tag of homologous gene from other strain
				dehydrogenase			
K-12 MG1655	*aroF*	-2.98	b2601	3-deoxy-7-phosphoheptulonate synthase, Tyr-sensitive	COG0722	1.35e-25	K8943_RS06435
K-12 MG1655	*cysJ*	-2.04	b2764	sulfite reductase, flavoprotein subunit	COG0369	7.39e-03	K8943_RS07060
K-12 MG1655	*cysI*	-3.28	b2763	sulfite reductase, hemoprotein subunit	COG0155	4.68e-07	K8943_RS07055
K-12 MG1655	*cysH*	-3.75	b2762	phosphoadenosine phosphosulfate reductase	COG0175	6.85e-04	K8943_RS07050
K-12 MG1655	*gltB*	-3.25	b3212	glutamate synthase subunit GltB	COG0069	2.14e-59	K8943_RS09945
K-12 MG1655	*gltD*	-3.01	b3213	glutamate synthase subunit GltD	COG0493	2.64e-27	K8943_RS09950
K-12 MG1655	*aceF*	-2.64	b0115	pyruvate dehydrogenase, E2 subunit	COG0508	1.09e-46	K8943_RS17255
K-12 MG1655	*lpd*	-1.9	b0116	lipoamide dehydrogenase	COG1249	2.05e-24	K8943_RS17260
K-12 MG1655	*aceE*	-3.25	b0114	pyruvate dehydrogenase E1 component	COG2609	1.71e-80	K8943_RS17250
K-12 MG1655	*cyoD*	-2.47	b0429	cytochrome bo3 subunit 4	COG3125	7.34e-07	K8943_RS19025
K-12 MG1655	*cyoB*	-2.55	b0431	cytochrome bo3 subunit 1	COG0843	3.81e-32	K8943_RS19035

strain	geneID	log2FoldChange	locus_tag	product	COGgene	padj	locus_tag of homologous gene from other strain
K-12 MG1655	*cyoC*	-3.0	b0430	cytochrome bo3 subunit 3	COG1845	1.30e-14	K8943_RS19030
K-12 MG1655	*ilvC*	-3.02	b3774	ketol-acid reductoisomerase (NADP(+))	COG0059	3.63e-08	K8943_RS12955
K-12 MG1655	*cysA*	-2.6	b2422	sulfate/thiosulfate ABC transporter ATP binding subunit	COG1118	5.36e-05	K8943_RS05640
K-12 MG1655	*cysM*	-1.57	b2421	cysteine synthase B	COG0031	8.18e-04	K8943_RS05635
K-12 MG1655	*cysW*	-2.97	b2423	sulfate/thiosulfate ABC transporter inner membrane subunit CysW	COG4208	4.77e-03	K8943_RS05645
K-12 MG1655	*cysD*	-1.77	b2752	sulfate adenylyltransferase subunit 2	COG0175	3.76e-02	K8943_RS07035
K-12 MG1655	*cysC*	-2.6	b2750	adenylyl-sulfate kinase	COG0529	1.08e-02	K8943_RS07025
K-12 MG1655	*cysN*	-1.96	b2751	sulfate adenylyltransferase subunit 1	COG2895	7.12e-05	K8943_RS07030
K-12 MG1655	*aroL*	-2.26	b0388	shikimate kinase 2	COG0703	6.48e-06	K8943_RS18820
K-12 MG1655	*serA*	-2.12	b2913	phosphoglycerate dehydrogenase	COG0111	3.60e-24	K8943_RS07850
K-12 MG1655	*ilvL*	-1.93	b3766	ilvXGMEDA operon leader peptide		3.39e-02	K8943_RS12915
K-12 MG1655	*icd*	-1.78	b1136	isocitrate dehydrogenase	COG0538	3.64e-27	K8943_RS22765

strain	geneID	log2FoldChange	locus_tag	product	COGgene	padj	locus_tag of homologous gene from other strain
K-12 MG1655	*ilvI*	-1.58	b0077	acetolactate synthase/acetohydroxybutanoate synthase, catalytic subunit	COG0028	1.22e-02	K8943_RS17035
K-12 MG1655	*fbaA*	0.42	b2925	fructose-bisphosphate aldolase class II	COG0191	4.86e-02	K8943_RS07895
K-12 MG1655	*aceA*	1.29	b4015	isocitrate lyase	COG2224	7.19e-16	K8943_RS14300
K-12 MG1655	*acnA*	1.59	b1276	aconitate hydratase 1	COG1048	4.42e-17	K8943_RS23755
K-12 MG1655	*fumC*	01.03	b1611	fumarase C	COG0114	3.48e-02	K8943_RS00825
K-12 MG1655	*frdB*	2.14	b4153	fumarate reductase iron-sulfur protein	COG0479	5.07e-04	K8943_RS15150
K-12 MG1655	*frdA*	2.39	b4154	fumarate reductase flavoprotein subunit	COG1053	1.01e-10	K8943_RS15155
K-12 MG1655	*fadM*	3.41	b0443	long-chain acyl-CoA thioesterase FadM	COG0824	3.86e-04	K8943_RS19095
K-12 MG1655	*csgF*	3.56	b1038	curli assembly component CsgF	ROG3787	3.47e-03	K8943_RS22285
K-12 MG1655	*csgG*	1.8	b1037	curli secretion channel	COG1462	1.45e-03	K8943_RS22280
K-12 MG1655	*csgE*	03.07	b1039	curli assembly component CsgE		2.06e-02	K8943_RS22290
K-12 MG1655	*csgD*	2.39	b1040	DNA-binding transcriptional	COG2197	1.57e-05	K8943_RS22295

strain	geneID	log2FoldChange	locus_tag	product	COGgene	padj	locus_tag of homologous gene from other strain
				dual regulator CsgD			
K-12 MG1655	*malG*	3.18	b4032	maltose ABC transporter membrane subunit MalG	COG3833	2.93e-03	K8943_RS14425
K-12 MG1655	*malE*	2.94	b4034	maltose ABC transporter periplasmic binding protein	COG2182	7.31e-08	K8943_RS14435
K-12 MG1655	*malF*	3.68	b4033	maltose ABC transporter membrane subunit MalF	COG1175	5.54e-04	K8943_RS14430
K-12 MG1655	*glpT*	3.99	b2240	sn-glycerol 3-phosphate:phosphate antiporter	COG2271	9.84e-15	K8943_RS04810
K-12 MG1655	*glpQ*	2.73	b2239	glycerophosphoryl diester phosphodiesterase GlpQ	COG0584	6.49e-11	K8943_RS04805
K-12 MG1655	*ytfQ*	4.12	b4227	galactofuranose ABC transporter periplasmic binding protein	COG1879	5.74e-14	K8943_RS15540
K-12 MG1655	*ytfR*	3.99	b4485	galactofuranose ABC transporter putative ATP binding subunit	COG1129	6.16e-09	K8943_RS15545
K-12 MG1655	*gatA*	3.82	b2094	galactitol-specific PTS enzyme IIA component	COG1762	1.70e-54	K8943_RS04000
K-12 MG1655	*gatB*	4.4	b2093	galactitol-specific PTS enzyme IIB component	COG3414	5.97e-17	K8943_RS03995
K-12 MG1655	*gatZ*	3.98	b2095	putative tagatose-1,6-bisphosphate aldolase 2	COG4573	2.54e-94	K8943_RS04005

strain	geneID	log2FoldChange	locus_tag	product	COGgene	padj	locus_tag of homologous gene from other strain
				chaperone			
K-12 MG1655	*gatY*	04.07	b2096	tagatose-1,6-bisphosphate aldolase 2	COG0191	3.26e-119	K8943_RS04010
K-12 MG1655	*fbaB*	0.92	b2097	fructose-bisphosphate aldolase class I	COG1830	1.56e-05	K8943_RS04015
K-12 MG1655	*glpA*	3.94	b2241	anaerobic glycerol-3-phosphate dehydrogenase subunit A	COG0578	3.67e-28	K8943_RS04815
K-12 MG1655	*glpC*	4.46	b2243	anaerobic glycerol-3-phosphate dehydrogenase subunit C	COG0247	1.03e-17	K8943_RS04825
K-12 MG1655	*glpB*	4.3	b2242	anaerobic glycerol-3-phosphate dehydrogenase subunit B	COG3075	1.43e-23	K8943_RS04820
K-12 MG1655	*glpK*	4.53	b3926	glycerol kinase	COG0554	1.28e-128	K8943_RS13815
K-12 MG1655	*glpF*	4.46	b3927	glycerol facilitator	COG0580	4.10e-57	K8943_RS13820
K-12 MG1655	*glpX*	3.1	b3925	fructose-1,6-bisphosphatase 2	COG1494	3.31e-19	K8943_RS13810
K-12 MG1655	*fadE*	4.54	b0221	acyl-CoA dehydrogenase	COG1960	7.97e-53	K8943_RS18225
K-12 MG1655	*lsrK*	4.83	b1511	autoinducer-2 kinase	COG1070	1.12e-26	
K-12 MG1655	*lsrR*	04.03	b1512	DNA-binding transcriptional	COG2390	7.39e-19	

strain	geneID	log2FoldChange	locus_tag	product	COGgene	padj	locus_tag of homologous gene from other strain
				repressor LsrR			
K-12 MG1655	*fadL*	4.87	b2344	long-chain fatty acid outer membrane channel/bacteriophage T2 receptor	COG2067	1.23e-14	K8943_RS05305
K-12 MG1655	*fadH*	4.94	b3081	2,4-dienoyl-CoA reductase	COG1902	5.05e-09	K8943_RS09315
K-12 MG1655	*fadJ*	3.38	b2341	3-hydroxyacyl-CoA dehydrogenase FadJ	COG1250	1.48e-19	K8943_RS05290
K-12 MG1655	*fadI*	4.95	b2342	3-ketoacyl-CoA thiolase FadI	COG0183	2.64e-31	K8943_RS05295
K-12 MG1655	*fadD*	5.19	b1805	long-chain-fatty-acid--CoA ligase	COG0318	1.20e-50	K8943_RS01810
K-12 MG1655	*gatD*	5.2	b2091	galactitol-1-phosphate 5-dehydrogenase	COG1063	1.13e-34	K8943_RS03985
K-12 MG1655	*csqR*	3.66	b3884	DNA-binding transcriptional dual regulator CsqR	COG1349	1.16e-08	K8943_RS13565
K-12 MG1655	*yihV*	5.3	b3883	6-deoxy-6-sulfofructose kinase	COG0524	1.15e-23	K8943_RS13570
K-12 MG1655	*mglB*	5.36	b2150	D-galactose/methyl-galactoside ABC transporter periplasmic binding protein	COG1879	1.55e-04	K8943_RS04280
K-12 MG1655	*mglA*	4.53	b2149	D-galactose/methyl-galactoside ABC transporter	COG1129	1.56e-03	K8943_RS04275

strain	geneID	log2FoldChange	locus_tag	product	COGgene	padj	locus_tag of homologous gene from other strain
				ATP binding subunit			
K-12 MG1655	*mglC*	3.11	b2148	D-galactose/methyl-galactoside ABC transporter membrane subunit	COG4211	1.44e-03	K8943_RS04270
K-12 MG1655	*treC*	5.67	b4239	trehalose-6-phosphate hydrolase	COG0366	7.28e-03	K8943_RS15595
K-12 MG1655	*fadA*	4.38	b3845	3-ketoacyl-CoA thiolase	COG0183	1.38e-12	K8943_RS13415
K-12 MG1655	*fadB*	5.73	b3846	multifunctional enoyl-CoA hydratase, 3-hydroxyacyl-CoA epimerase, Delta(3)-cis-Delta(2)-trans-enoyl-CoA isomerase, L-3-hydroxyacyl-CoA dehydrogenase	COG1250	5.05e-27	K8943_RS13420
K-12 MG1655	*astE*	4.96	b1744	succinylglutamate desuccinylase	COG2988	3.70e-35	K8943_RS01500
K-12 MG1655	*astD*	5.13	b1746	aldehyde dehydrogenase	COG1012	2.65e-53	K8943_RS01510
K-12 MG1655	*astC*	5.29	b1748	succinylornithine transaminase	COG4992	4.14e-68	K8943_RS01520
K-12 MG1655	*astB*	4.93	b1745	N-succinylarginine dihydrolase	COG3724	7.35e-44	K8943_RS01505
K-12 MG1655	*astA*	5.73	b1747	arginine N-succinyltransferase	COG3138	1.05e-43	K8943_RS01515

strain	geneID	log2FoldChange	locus_tag	product	COGgene	padj	locus_tag of homologous gene from other strain
K-12 MG1655	*ydcT*	4.58	b1441	putative ABC transporter ATP-binding protein YdcT	COG3842	3.53e-16	K8943_RS00135
K-12 MG1655	*ydcU*	4.47	b1442	putative ABC transporter membrane subunit YdcU	COG1176	1.37e-10	K8943_RS00140
K-12 MG1655	*ydcV*	4.42	b1443	putative ABC transporter membrane subunit YdcV	COG1177	2.79e-13	K8943_RS00145
K-12 MG1655	*patD*	4.2	b1444	gamma-aminobutyraldehyde dehydrogenase	COG1012	1.08e-34	K8943_RS00150
K-12 MG1655	*ydcS*	5.84	b1440	putative ABC transporter periplasmic binding protein/polyhydroxybutyrate synthase	COG0687	3.15e-36	K8943_RS00130
K-12 MG1655	*xylF*	6.5	b3566	xylose ABC transporter periplasmic binding protein	COG4213	1.06e-03	K8943_RS11810
K-12 MG1655	*malK*	6.69	b4035	maltose ABC transporter ATP binding subunit	COG3839	7.12e-05	K8943_RS14440
K-12 MG1655	*lamB*	3.13	b4036	maltose outer membrane channel/phage lambda receptor protein	COG4580	3.32e-07	K8943_RS14445
K-12 MG1655	*ompL*	7.29	b3875	putative outer membrane porin L		6.07e-05	
K-12 MG1655	*yjcH*	7.46	b4068	DUF485 domain-containing inner membrane protein YjcH	COG3162	6.72e-06	K8943_RS14685

strain	geneID	log2FoldChange	locus_tag	product	COGgene	padj	locus_tag of homologous gene from other strain
K-12 MG1655	*lsrC*	8.56	b1514	Autoinducer-2 ABC transporter membrane subunit LsrC	COG1172	8.43e-08	
K-12 MG1655	*lsrA*	6.58	b1513	Autoinducer-2 ABC transporter ATP binding subunit	COG1129	6.14e-19	
K-12 MG1655	*lsrD*	06.03	b1515	Autoinducer-2 ABC transporter membrane subunit LsrD	COG1172	2.34e-12	
K-12 MG1655	*lsrB*	6.81	b1516	Autoinducer-2 ABC transporter periplasmic binding protein	COG1879	8.26e-21	
K-12 MG1655	*yihO*	7.89	b3876	putative sulfoquinovose transporter	COG2211	2.03e-12	
K-12 MG1655	*yihS*	7.89	b3880	sulfoquinovose isomerase	COG2942	5.93e-40	
K-12 MG1655	*yihQ*	08.01	b3878	sulfoquinovosidase	COG1501	1.08e-46	
K-12 MG1655	*yihU*	8.84	b3882	3-sulfolactaldehyde reductase	COG2084	2.44e-08	K8943_RS13575
K-12 MG1655	*yihP*	9.23	b3877	putative 2,3-dihydroxypropane-1-sulfonate export protein	COG2211	4.29e-09	
K-12 MG1655	*yihT*	9.74	b3881	6-deoxy-6-sulfofructose-1-phosphate aldolase	COG3684	4.51e-10	K8943_RS13580

strain	geneID	log2FoldChange	locus_tag	product	COGgene	padj	locus_tag of homologous gene from other strain
K-12 MG1655	*yihR*	10.14	b3879	putative sulfoquinovose mutarotase YihR	COG2017	1.01e-10	
K-12 MG1655	*prpE*	8.82	b0335	propionyl-CoA synthetase	COG0365	1.88e-64	K8943_RS18640
K-12 MG1655	*prpD*	11.03	b0334	2-methylcitrate dehydratase	COG2079	1.35e-36	K8943_RS18635
K-12 MG1655	*prpC*	11.25	b0333	2-methylcitrate synthase	COG0372	6.62e-26	K8943_RS18630
K-12 MG1655	*prpB*	12.24	b0331	2-methylisocitrate lyase	COG2513	1.20e-30	K8943_RS18625
Nissle 1917	*tauA*	-7.31	K8943_RS18720	taurine ABC transporter substrate-binding protein	COG4521	1.66e-05	b0365
Nissle 1917	*tauD*	-2.78	K8943_RS18735	taurine dioxygenase	COG2175	1.12e-02	b0368
Nissle 1917	*tauC*	-3.04	K8943_RS18730	taurine ABC transporter permease TauC	COG0600	2.41e-02	b0367
Nissle 1917	*grcA*	-6.72	K8943_RS06325	autonomous glycyl radical cofactor GrcA	COG3445	3.90e-130	b2579
Nissle 1917	*ilvC*	-6.59	K8943_RS12955	ketol-acid reductoisomerase	COG0059	2.12e-108	b3774
Nissle 1917	*tyrA*	-5.66	K8943_RS06430	bifunctional chorismate mutase/prephenate dehydrogenase	COG1605	5.44e-114	b2600
Nissle 1917	*aroF*	-6.37	K8943_RS06435	3-deoxy-7-phosphoheptulonate synthase	COG0722	3.00e-165	b2601

strain	geneID	log2FoldChange	locus_tag	product	COGgene	padj	locus_tag of homologous gene from other strain
				AroF			
Nissle 1917	*btsT*	-6.35	K8943_RS16375	pyruvate/proton symporter BtsT	COG1966	3.81e-76	b4354
Nissle 1917	*ynfM*	-5.89	K8943_RS00735	MFS transporter	COG0477	1.65e-38	b1596
Nissle 1917	*sbp*	-5.27	K8943_RS13770	sulfate/thiosulfate ABC transporter substrate-binding protein Sbp	COG1613	1.26e-49	b3917
Nissle 1917	*ybfA*	-5.19	K8943_RS20190	YbfA family protein	COG5664	9.30e-10	b0699
Nissle 1917	*nirD*	-5.06	K8943_RS10715	nitrite reductase small subunit NirD	COG2146	1.09e-02	b3366
Nissle 1917	*nirB*	-4.81	K8943_RS10710	NADPH-nitrite reductase large subunit	COG1251	3.99e-22	b3365
Nissle 1917	*metE*	-4.82	K8943_RS13240	5-methyltetrahydropteroyltriglutamate--homocysteine S-methyltransferase	COG0620	1.25e-224	b3829
Nissle 1917	*tyrP*	-4.63	K8943_RS02615	tyrosine transporter TyrP	COG0814	2.10e-18	b1907
Nissle 1917	*metF*	-4.55	K8943_RS13915	methylenetetrahydrofolate reductase	COG0685	2.68e-85	b3941
Nissle 1917	*ndh*	-4.55	K8943_RS2	NADH-quinone	COG1252	1.14e-62	b1109

strain	geneID	log2FoldChange	locus_tag	product	COGgene	padj	locus_tag of homologous gene from other strain
			2630	dehydrogenase			
Nissle 1917	*thiS*	-3.98	K8943_RS14175	sulfur carrier protein ThiS	COG2104	1.20e-17	b4407
Nissle 1917	*thiF*	-4.1	K8943_RS14180	thiazole biosynthesis adenylyltransferase ThiF	COG0476	8.72e-48	b3992
Nissle 1917	*thiG*	-4.24	K8943_RS14170	thiazole synthase	COG2022	2.42e-62	b3991
Nissle 1917	*thiC*	-4.38	K8943_RS14190	phosphomethylpyrimidine synthase ThiC	COG0422	1.20e-138	b3994
Nissle 1917	*thiE*	-4.49	K8943_RS14185	thiamine phosphate synthase	COG0352	5.71e-55	b3993
Nissle 1917	*thiH*	-3.55	K8943_RS14165	2-iminoacetate synthase ThiH	COG1060	1.42e-69	b3990
Nissle 1917	*fliT*	-3.79	K8943_RS02715	flagella biosynthesis regulatory protein FliT	ROG0925	2.69e-13	b1926
Nissle 1917	*fliD*	-3.07	K8943_RS02705	flagellar filament capping protein FliD	COG1345	4.00e-54	b1924
Nissle 1917	*fliS*	-2.78	K8943_RS02710	flagellar export chaperone FliS	COG1516	1.19e-10	b1925
Nissle 1917	*cysN*	-3.72	K8943_RS07030	sulfate adenylyltransferase subunit CysN	COG2895	7.72e-109	b2751

strain	geneID	log2FoldChange	locus_tag	product	COGgene	padj	locus_tag of homologous gene from other strain
Nissle 1917	*cysD*	-3.64	K8943_RS07035	sulfate adenylyltransferase subunit CysD	COG0175	4.66e-95	b2752
Nissle 1917	*cysC*	-3.16	K8943_RS07025	adenylyl-sulfate kinase	COG0529	5.12e-34	b2750
Nissle 1917	*fliK*	-2.69	K8943_RS02800	flagellar hook-length control protein FliK	COG3144	4.45e-21	b1943
Nissle 1917	*fliF*	-2.69	K8943_RS02775	flagellar basal-body MS-ring/collar protein FliF	COG1766	1.48e-28	b1938
Nissle 1917	*fliQ*	-2.73	K8943_RS02830	flagellar biosynthesis protein FliQ	COG1987	1.47e-02	b1949
Nissle 1917	*fliL*	-1.99	K8943_RS02805	flagellar basal body-associated protein FliL	COG1580	3.14e-08	b1944
Nissle 1917	*fliJ*	-2.83	K8943_RS02795	flagellar export protein FliJ	COG2882	1.40e-06	b1942
Nissle 1917	*fliH*	-2.84	K8943_RS02785	flagellar assembly protein FliH	COG1317	7.69e-18	b1940
Nissle 1917	*fliO*	-1.99	K8943_RS02820	flagellar biosynthetic protein FliO	COG3190	5.08e-04	b1947
Nissle 1917	*fliI*	-3.04	K8943_RS02790	flagellar protein export ATPase FliI	COG1157	3.33e-26	b1941
Nissle 1917	*fliG*	-3.05	K8943_RS02780	flagellar motor switch protein FliG	COG1536	1.15e-28	b1939

strain	geneID	log2FoldChange	locus_tag	product	COGgene	padj	locus_tag of homologous gene from other strain
Nissle 1917	*fliM*	-2.53	K8943_RS02810	flagellar motor switch protein FliM	COG1868	6.13e-19	b1945
Nissle 1917	*fliP*	-2.2	K8943_RS02825	flagellar type III secretion system pore protein FliP	COG1338	4.09e-05	b1948
Nissle 1917	*fliN*	-3.68	K8943_RS02815	flagellar motor switch protein FliN	COG1886	1.68e-10	b1946
Nissle 1917	*cysM*	-2.02	K8943_RS05635	cysteine synthase B	COG0031	1.32e-12	b2421
Nissle 1917	*cysP*	-3.66	K8943_RS05655	thiosulfate/sulfate ABC transporter substrate-binding protein CysP	COG4150	1.71e-88	b2425
Nissle 1917	*cysA*	-3.06	K8943_RS05640	sulfate/thiosulfate ABC transporter ATP-binding protein CysA	COG1118	2.95e-62	b2422
Nissle 1917	*cysW*	-3.02	K8943_RS05645	sulfate/thiosulfate ABC transporter permease CysW	COG4208	2.96e-43	b2423
Nissle 1917	*ilvL*	-3.52	K8943_RS12915	ilv operon leader peptide		1.85e-02	b3766
Nissle 1917	*flgC*	-3.38	K8943_RS22460	flagellar basal body rod protein FlgC	COG1558	7.24e-21	b1074
Nissle 1917	*flgL*	-2.71	K8943_RS22505	flagellar hook-associated protein FlgL	ROG2760	2.08e-44	b1083
Nissle 1917	*flgF*	-2.84	K8943_RS2	flagellar basal-body rod	COG4787	9.16e-35	b1077

strain	geneID	log2FoldChange	locus_tag	product	COGgene	padj	locus_tag of homologous gene from other strain
			2475	protein FlgF			
Nissle 1917	*flgB*	-2.8	K8943_RS22455	flagellar basal body rod protein FlgB	COG1815	7.74e-27	b1073
Nissle 1917	*flgD*	-2.73	K8943_RS22465	flagellar hook assembly protein FlgD	COG1843	2.19e-44	b1075
Nissle 1917	*flgG*	-2.87	K8943_RS22480	flagellar basal-body rod protein FlgG	COG4786	4.23e-38	b1078
Nissle 1917	*flgK*	-2.88	K8943_RS22500	flagellar hook-associated protein FlgK	COG1256	1.26e-55	b1082
Nissle 1917	*flgH*	-2.88	K8943_RS22485	flagellar basal body L-ring protein FlgH	COG2063	2.97e-22	b1079
Nissle 1917	*flgE*	-2.47	K8943_RS22470	flagellar hook protein FlgE	COG1749	6.45e-53	b1076
Nissle 1917	*flgI*	-2.96	K8943_RS22490	flagellar basal body P-ring protein FlgI	COG1706	1.11e-31	b1080
Nissle 1917	*flgJ*	-2.56	K8943_RS22495	flagellar assembly peptidoglycan hydrolase FlgJ	COG3951	7.11e-23	b1081
Nissle 1917	*cysI*	-2.81	K8943_RS07055	assimilatory sulfite reductase (NADPH) hemoprotein subunit	COG0155	4.30e-46	b2763
Nissle 1917	*cysH*	-3.31	K8943_RS07050	phosphoadenosine phosphosulfate reductase	COG0175	6.72e-35	b2762

strain	geneID	log2FoldChange	locus_tag	product	COGgene	padj	locus_tag of homologous gene from other strain
Nissle 1917	*cysJ*	-2.88	K8943_RS07060	NADPH-dependent assimilatory sulfite reductase flavoprotein subunit	COG0369	4.34e-53	b2764
Nissle 1917	*aroL*	-3.12	K8943_RS18820	shikimate kinase AroL	COG0703	1.27e-25	b0388
Nissle 1917	*serA*	-3.08	K8943_RS07850	phosphoglycerate dehydrogenase	COG0111	3.45e-72	b2913
Nissle 1917	*fliZ*	-2.52	K8943_RS02690	flagella biosynthesis regulatory protein FliZ		4.65e-12	b1921
Nissle 1917	*fliA*	-2.58	K8943_RS02695	RNA polymerase sigma factor FliA	COG1191	2.21e-44	b1922
Nissle 1917	*flgN*	-2.03	K8943_RS22440	flagella biosynthesis chaperone FlgN	COG3418	2.42e-11	b1070
Nissle 1917	*flgA*	-3.06	K8943_RS22450	flagellar basal body P-ring formation chaperone FlgA	COG1261	8.75e-14	b1072
Nissle 1917	*flgM*	-2.12	K8943_RS22445	flagellar biosynthesis anti-sigma factor FlgM	COG2747	4.08e-09	b1071
Nissle 1917	*ilvN*	-2.91	K8943_RS17040	acetolactate synthase small subunit	COG0440	5.67e-04	b0078
Nissle 1917	*ilvI*	-1.14	K8943_RS17035	acetolactate synthase 3 large subunit	COG0028	3.84e-04	b0077
Nissle 1917	*gltB*	-2.54	K8943_RS09945	glutamate synthase large subunit	COG0069	1.79e-35	b3212

strain	geneID	log2FoldChange	locus_tag	product	COGgene	padj	locus_tag of homologous gene from other strain
Nissle 1917	*gltD*	-2.86	K8943_RS09950	glutamate synthase subunit GltD	COG0493	3.10e-30	b3213
Nissle 1917	*aceF*	-2.13	K8943_RS17255	pyruvate dehydrogenase complex dihydrolipoyllysine-residue acetyltransferase	COG0508	7.95e-41	b0115
Nissle 1917	*aceE*	-2.12	K8943_RS17250	pyruvate dehydrogenase (acetyl-transferring), homodimeric type	COG2609	5.58e-49	b0114
Nissle 1917	*ilvG*	-1.62	K8943_RS12925	acetolactate synthase 2 catalytic subunit	COG0028	9.81e-05	
Nissle 1917	*ilvD*	-1.74	K8943_RS12940	dihydroxy-acid dehydratase	COG0129	1.03e-14	b3771
Nissle 1917	*ilvA*	-1.81	K8943_RS12945	threonine ammonia-lyase, biosynthetic	COG1171	1.60e-11	b3772
Nissle 1917	*ilvE*	-1.82	K8943_RS12935	branched-chain-amino-acid transaminase	COG0115	8.21e-10	b3770
Nissle 1917	*fliE*	-1.74	K8943_RS02770	flagellar hook-basal body complex protein FliE	COG1677	1.36e-04	b1937
Nissle 1917	*zwf*	-0.77	K8943_RS02050	glucose-6-phosphate dehydrogenase	COG0364	7.44e-03	b1852
Nissle 1917	*icd*	0.6	K8943_RS22765	NADP-dependent isocitrate dehydrogenase	COG0538	1.33e-05	b1136

strain	geneID	log2FoldChange	locus_tag	product	COGgene	padj	locus_tag of homologous gene from other strain
Nissle 1917	*acnA*	1.27	K8943_RS23755	aconitate hydratase AcnA	COG1048	7.71e-04	b1276
Nissle 1917	*ilvB*	1.37	K8943_RS12455	acetolactate synthase large subunit	COG0028	3.77e-02	b3671
Nissle 1917	*fbp*	1.91	K8943_RS15560	class 1 fructose-bisphosphatase	COG0158	2.84e-12	b4232
Nissle 1917	*acnB*	2.23	K8943_RS17270	bifunctional aconitate hydratase 2/2-methylisocitrate dehydratase	COG1049	7.78e-44	b0118
Nissle 1917	*cyoC*	1.66	K8943_RS19030	cytochrome o ubiquinol oxidase subunit III	COG1845	1.08e-08	b0430
Nissle 1917	*cyoB*	1.96	K8943_RS19035	cytochrome o ubiquinol oxidase subunit I	COG0843	2.93e-25	b0431
Nissle 1917	*cyoA*	2.36	K8943_RS19040	cytochrome o ubiquinol oxidase subunit II	COG1622	5.65e-37	b0432
Nissle 1917	*cyoD*	1.69	K8943_RS19025	cytochrome o ubiquinol oxidase subunit IV	COG3125	1.49e-06	b0429
Nissle 1917	*cyoE*	01.07	K8943_RS19020	heme o synthase	COG0109	2.00e-07	b0428
Nissle 1917	*fadD*	2.6	K8943_RS01810	long-chain-fatty-acid--CoA ligase FadD	COG0318	1.89e-15	b1805
Nissle 1917	*malE*	2.81	K8943_RS14435	maltose/maltodextrin ABC transporter substrate-binding	COG2182	9.76e-18	b4034

strain	geneID	log2FoldChange	locus_tag	product	COGgene	padj	locus_tag of homologous gene from other strain
				protein MalE			
Nissle 1917	*malF*	1.66	K8943_RS14430	maltose ABC transporter permease MalF	COG1175	4.80e-03	b4033
Nissle 1917	*malG*	2.27	K8943_RS14425	maltose ABC transporter permease MalG	COG3833	4.26e-03	b4032
Nissle 1917	*glcA*	2.88	K8943_RS08680	glycolate permease GlcA	COG1620	1.61e-02	b2975
Nissle 1917	*ppsA*	2.94	K8943_RS01295	phosphoenolpyruvate synthase	COG0574	1.35e-49	b1702
Nissle 1917	*lamB*	03.08	K8943_RS14445	maltoporin LamB	COG4580	1.16e-17	b4036
Nissle 1917	*malK*	2.55	K8943_RS14440	maltose/maltodextrin ABC transporter ATP-binding protein MalK	COG3839	6.34e-05	b4035
Nissle 1917	*sthA*	3.23	K8943_RS14025	Si-specific NAD(P)(+) transhydrogenase	COG1249	1.12e-63	b3962
Nissle 1917	*fadI*	3.22	K8943_RS05295	acetyl-CoA C-acyltransferase FadI	COG0183	2.73e-03	b2342
Nissle 1917	*fadJ*	3.33	K8943_RS05290	fatty acid oxidation complex subunit alpha FadJ	COG1250	1.79e-20	b2341
Nissle 1917	*pckA*	3.38	K8943_RS10870	phosphoenolpyruvate carboxykinase (ATP)	COG1866	6.30e-73	b3403

strain	geneID	log2FoldChange	locus_tag	product	COGgene	padj	locus_tag of homologous gene from other strain
Nissle 1917	*fadL*	3.43	K8943_RS05305	long-chain fatty acid transporter FadL	COG2067	5.88e-27	b2344
Nissle 1917	*ydcV*	3.75	K8943_RS00145	ABC transporter permease	COG1177	6.71e-05	b1443
Nissle 1917	*ydcS*	3.53	K8943_RS00130	ABC transporter substrate-binding protein	COG0687	1.56e-12	b1440
Nissle 1917	*ydcU*	3.85	K8943_RS00140	ABC transporter permease	COG1176	7.33e-04	b1442
Nissle 1917	*patD*	3.45	K8943_RS00150	aminobutyraldehyde dehydrogenase	COG1012	9.24e-15	b1444
Nissle 1917	*xylF*	3.93	K8943_RS11810	D-xylose ABC transporter substrate-binding protein	COG4213	4.58e-03	b3566
Nissle 1917	*fadH*	4.56	K8943_RS09315	NADPH-dependent 2,4-dienoyl-CoA reductase	COG1902	2.79e-07	b3081
Nissle 1917	*ytfQ*	4.92	K8943_RS15540	galactofuranose ABC transporter substrate-binding protein YtfQ	COG1879	1.49e-10	b4227
Nissle 1917	*ytfR*	2.37	K8943_RS15545	sugar ABC transporter ATP-binding protein	COG1129	3.66e-03	b4485
Nissle 1917	*fumC*	3.83	K8943_RS00825	class II fumarate hydratase	COG0114	7.13e-23	b1611
Nissle 1917	*fadE*	5.25	K8943_RS18225	acyl-CoA dehydrogenase FadE	COG1960	8.68e-27	b0221

strain	geneID	log2FoldChange	locus_tag	product	COGgene	padj	locus_tag of homologous gene from other strain
Nissle 1917	*sdhC*	5.4	K8943_RS20255	succinate dehydrogenase cytochrome b556 subunit	COG2009	2.11e-39	b0721
Nissle 1917	*cds-WP_000426105.1*	5.52	K8943_RS21985	CPBP family intramembrane metalloprotease	COG1266	7.81e-12	
Nissle 1917	*prpB*	4.96	K8943_RS18625	methylisocitrate lyase	COG2513	3.95e-09	b0331
Nissle 1917	*prpD*	5.57	K8943_RS18635	2-methylcitrate dehydratase	COG2079	1.65e-12	b0334
Nissle 1917	*prpE*	3.94	K8943_RS18640	propionate--CoA ligase	COG0365	1.26e-09	b0335
Nissle 1917	*prpC*	3.98	K8943_RS18630	2-methylcitrate synthase	COG0372	4.25e-08	b0333
Nissle 1917	*aceB*	5.73	K8943_RS14295	malate synthase A	COG2225	6.91e-74	b4014
Nissle 1917	*aceA*	4.52	K8943_RS14300	isocitrate lyase	COG2224	7.45e-75	b4015
Nissle 1917	*glcF*	3.65	K8943_RS08695	glycolate oxidase subunit GlcF	COG0247	5.51e-14	b4467
Nissle 1917	*glcG*	3.64	K8943_RS08690	heme-binding protein	COG3193	2.74e-13	b2977
Nissle 1917	*glcB*	3.18	K8943_RS08685	malate synthase G	COG2225	1.42e-35	b2976

strain	geneID	log2FoldChange	locus_tag	product	COGgene	padj	locus_tag of homologous gene from other strain
Nissle 1917	*glcE*	5.19	K8943_RS08700	glycolate oxidase subunit GlcE	COG0277	4.90e-14	b4468
Nissle 1917	*mglC*	4.84	K8943_RS04270	galactose/methyl galactoside ABC transporter permease MglC	COG4211	1.44e-18	b2148
Nissle 1917	*mglB*	5.85	K8943_RS04280	galactose/glucose ABC transporter substrate-binding protein MglB	COG1879	6.58e-40	b2150
Nissle 1917	*mglA*	06.02	K8943_RS04275	galactose/methyl galactoside ABC transporter ATP-binding protein MglA	COG1129	2.97e-12	b2149
Nissle 1917	*cds-WP_000629094.1*	4.41	K8943_RS08375	N-acetylmannosamine kinase	COG1940	1.47e-06	
Nissle 1917	*cds-WP_000865295.1*	1.82	K8943_RS08355	N-acetylneuraminate epimerase	COG3055	3.21e-02	
Nissle 1917	*cds-WP_001149834.1*	06.04	K8943_RS08380	N-acetylneuraminate lyase	COG0329	8.24e-07	
Nissle 1917	*yjfN*	6.12	K8943_RS15320	DUF1471 family protease activator YjfN		6.23e-04	b4188
Nissle 1917	*argT*	6.48	K8943_RS05140	lysine/arginine/ornithine ABC transporter substrate-binding protein ArgT	COG0834	7.21e-32	b2310

strain	geneID	log2FoldChange	locus_tag	product	COGgene	padj	locus_tag of homologous gene from other strain
Nissle 1917	*acs*	6.55	K8943_RS14690	acetate--CoA ligase	COG0365	6.90e-188	b4069
Nissle 1917	*actP*	07.04	K8943_RS14680	cation/acetate symporter ActP	COG4147	2.31e-66	b4067
Nissle 1917	*fadA*	6.71	K8943_RS13415	acetyl-CoA C-acyltransferase FadA	COG0183	1.26e-12	b3845
Nissle 1917	*fadB*	7.12	K8943_RS13420	fatty acid oxidation complex subunit alpha FadB	COG1250	1.02e-27	b3846
Nissle 1917	*cds-WP_001529506.1*	7.67	K8943_RS18050	hypothetical protein		7.97e-12	
Nissle 1917	*astE*	6.37	K8943_RS01500	succinylglutamate desuccinylase	COG2988	3.30e-60	b1744
Nissle 1917	*astB*	6.83	K8943_RS01505	N-succinylarginine dihydrolase	COG3724	1.04e-56	b1745
Nissle 1917	*astD*	6.87	K8943_RS01510	succinylglutamate-semialdehyde dehydrogenase	COG1012	1.94e-48	b1746
Nissle 1917	*astA*	7.95	K8943_RS01515	arginine N-succinyltransferase	COG3138	3.63e-30	b1747
Nissle 1917	*astC*	8.31	K8943_RS01520	succinylornithine/acetylornithine transaminase	COG4992	6.51e-52	b1748
Nissle 1917	*cds-WP_0015*	8.48	K8943_RS21990	hypothetical protein	COG4097	7.37e-08	

strain	geneID	log2FoldChange	locus_tag	product	COGgene	padj	locus_tag of homologous gene from other strain
	28902.1						
